# Cardiac Involvement by Human Herpesvirus 8-Positive Diffuse Large B-Cell Lymphoma: An Unusual Presentation in a Patient with Human Immunodeficiency Virus

**DOI:** 10.1155/2022/1298121

**Published:** 2022-01-17

**Authors:** Elena M. Fenu, Michael W. Beaty, Tiffany E. O'Neill, Stacey S. O'Neill

**Affiliations:** Atrium Wake Forest Baptist Health, Department of Pathology, Winston-Salem, NC, USA

## Abstract

Human immunodeficiency virus (HIV) infection predisposes patients to the development of lymphomas, both due to immune suppression and coinfection with viruses with oncogenic potential. Coinfection with human herpesvirus 8 (HHV8) in particular has been associated with the development of aggressive lymphomas, including primary effusion lymphoma and diffuse large B-cell lymphoma (DLBCL). Herein, we report an unusual case of HHV8-positive DLBCL with extensive cardiac involvement which was diagnosed at autopsy in a patient with long-standing untreated HIV infection.

## 1. Introduction

Patients with human immunodeficiency virus (HIV) infection are at increased risk for infection and malignancy due to impaired cellular immunity. Prior to the advent of modern antiretroviral therapy (ART), HIV-positive individuals had a risk of acquiring non-Hodgkin lymphoma that was 25- to 150-times higher than that of the general population [1]. Although treatment with ART has greatly improved life expectancy and decreased cancer risk, lymphoma remains a leading cause of mortality in individuals with HIV infection [2].

Due to immune suppression and immune dysregulation, HIV-infected individuals are susceptible to coinfection with other viruses including those with oncogenic potential such as human herpesvirus 8 (HHV8) and Epstein-Barr virus (EBV). Such viral-associated lymphoproliferative disorders include multicentric Castleman disease, primary effusion lymphoma (PEL), and HHV8-positive diffuse large B-cell lymphoma (DLBCL). Herein, we report a case of an unusual presentation of a rare and aggressive lymphoma in a young patient due to coinfection with HIV and HHV8.

A 38-year-old male with a history of known, but untreated, HIV for approximately 9 years, was found face down deceased at his residence when a welfare check was conducted. Per his significant other, he had multiple episodes of fainting on the day of his death. An autopsy was performed.

**Figure 1:**
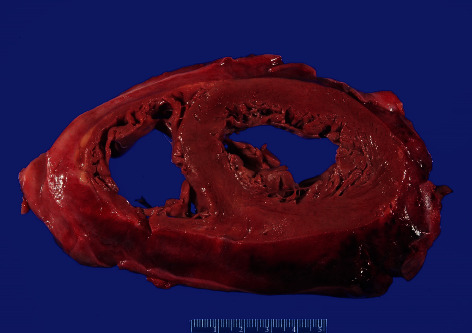
A photograph of a cross-section of the heart with thickened and gelatinous-appearing epicardium.

At autopsy, the heart was grossly abnormal, with increased size (900 grams) and a thickened, red-pink gelatinous epicardial surface (Figure 1). Numerous adhesions between the pericardial and epicardial surfaces were present. Of note, there was no measurable pericardial effusion. Focal firmness and tan discoloration of the right atrium were evident, without overt increase in myocardial wall thickness. Heart valves were unremarkable, and coronary arteries were widely patent. There was diffuse lymphadenopathy, including prominent intraparenchymal pulmonary, mediastinal, perigastric, and peripancreatic lymph nodes. Splenomegaly (710 grams) was present. No skin lesions or other significant gross abnormalities were identified. No pleural effusion or ascites was detected.

**Figure 2:**
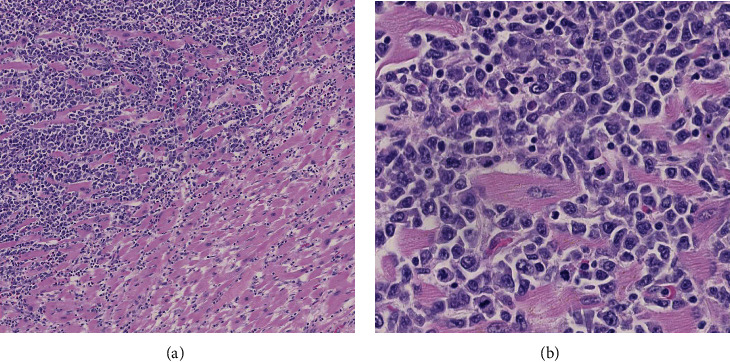
Histologic sections of the heart showed infiltration by mitotically active, pleomorphic cells, with associated myonecrosis. (a) and (b)H&E (hematoxylin and eosin) at 10× and 40× magnification, respectively.

On microscopic examination, sections of the right atrium of the heart demonstrated transmural infiltration by large pleomorphic cells with a plasmablastic appearance with prominent nucleoli and high nuclear to cytoplasmic ratio. Abundant mitoses were present. There was extensive disruption of normal cardiac architecture and associated myocyte injury (Figure 2). The right and left ventricles were uninvolved. Sections of the thickened epicardium showed chronic pericarditis without an atypical large lymphoid cell infiltrate. Representative sections of peripancreatic and perigastric lymph nodes showed sheets of similar-appearing neoplastic cells extending into adipose tissue. Sections of uninvolved pulmonary lymph nodes were significant for a prominent sinusoidal and capsular expansion of small mature plasma cells. The spleen showed increased plasma cells around central arterioles, but was negative for infiltration by the large pleomorphic lymphoid cells.

**Figure 3:**
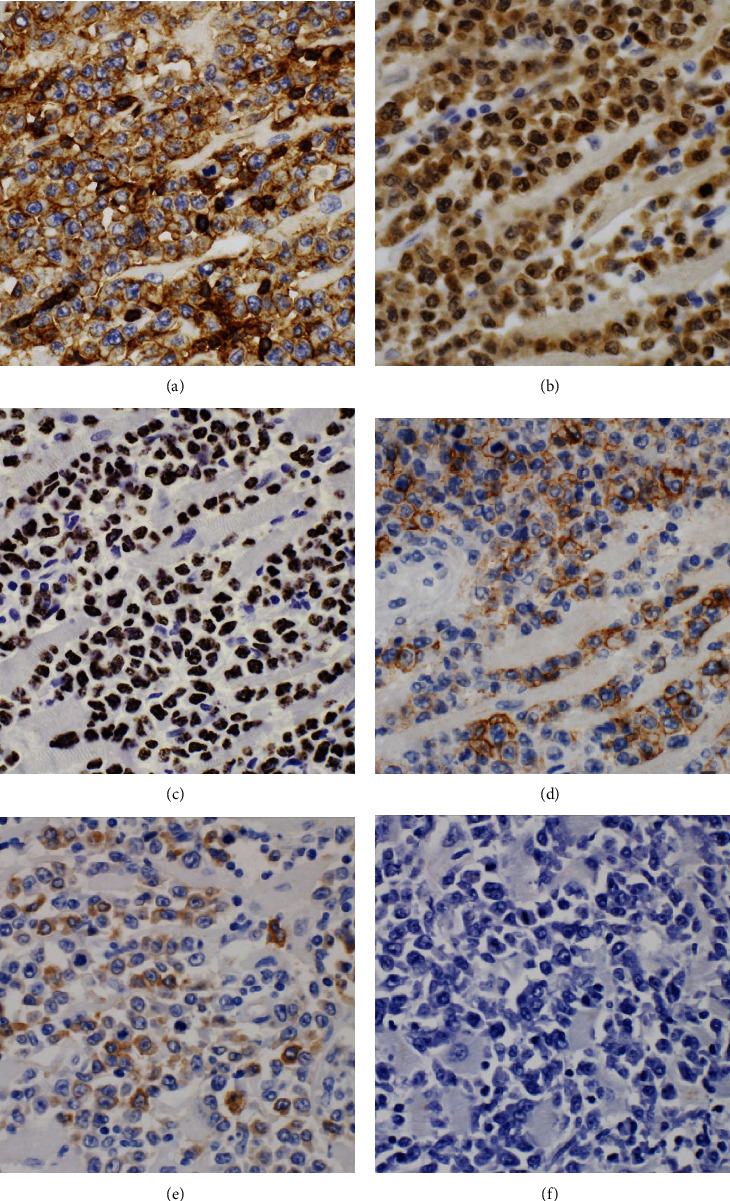
Immunohistochemical staining patterns demonstrated the neoplastic cells were positive for CD45 (a), MUM1 (b), HHV8 (c), CD20, subset (d), and CD30, subset (e). EBV in situ hybridization was negative (f).

**Figure 4:**
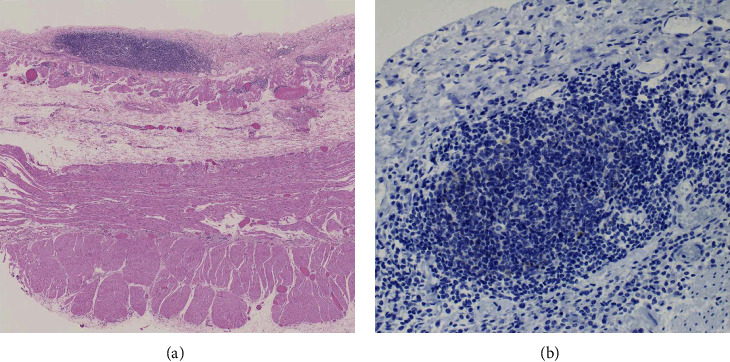
Histologic section of the pericardium, showing pericarditis without a large cell infiltrate (a) and absence of staining for HHV8 (b).

By immunohistochemistry, the large pleomorphic cells within the right atrium stained positive for CD45 and MUM1 and were negative for CD138, CD79a, PAX5, CD19, BCL6, MYC, CD56, and CD2 (Figure 3). A subset of the cells stained dimly positive for CD20 and CD30. Immunohistochemistry for HHV8-associated latent protein was strongly and diffusely positive in the neoplastic cells. In situ hybridization for EBV-encoded small RNA (EBER) was negative. Light chain in situ hybridization showed variable weak staining for kappa light chains in the large neoplastic cells; lambda light chain was negative. Of note, flow cytometric analysis was not possible due to lack of fresh tissue procured at the time of autopsy. Sections of pericardium confirmed the absence of a significant HHV8-positive large cell infiltrate (Figure 4), supporting a reactive pericarditis.

Together, the findings were indicative of an aggressive HHV8-associated lymphoproliferative disorder, with the leading differential diagnoses including HHV8-positive DLBCL and extracavitary variant of PEL. These two entities can show overlapping morphologic and immunophenotypic features, and distinguishing them can be challenging [3]. In addition to HHV8, PEL typically expresses CD45, CD138, CD38, MUM1, CD30, and EMA, but lacks B-cell antigens CD19, CD20, and CD79a. Surface and cytoplasmic light chain expression is typically absent. Of note, the extracavitary variant of PEL can show some B-cell antigen and/or immunoglobulin expression. Importantly, PEL in HIV-positive patients is invariably positive for EBV [4]. In HHV8-positive DLBCL, the neoplastic cells are typically positive for MUM1 with variable expression of CD45 and B-cell markers CD20, PAX5, and without expression of CD138, CD79a, or EBER. Restricted light chain expression, frequently lambda, is often seen. In the current case, the lack of EBV and CD138 in conjunction with the presence of diffuse lymphadenopathy favors the diagnosis of HHV8-positive DLBCL, not otherwise specified (NOS) in this patient with long-standing untreated HIV infection. The extensive cardiac involvement was deemed to have directly caused cardiac arrest and death.

Cardiac involvement by lymphoma is unusual. A review of case reports published between 2009 and 2019 found only 158 cases of lymphoma involving the heart, of which 101 were defined as primary cardiac lymphomas (lymphomas with only cardiac involvement) [5]. In this review, the most common lymphoma to involve the heart was DLBCL (88 out of 101 cases), and the right atrium and right ventricle were most frequently involved [6]. Lymphomas may also involve the heart after becoming widely disseminated [7]. Cardiac involvement by nonprimary disease is rare, and a population series reviewing over 12,000 autopsies found heart metastases in 1.23% of cases, of which the most commonly encountered were lung, lymphoma, breast, leukemia, stomach, melanoma, liver, and colon [8]. In cases of cardiac involvement by lymphoma, common presenting symptoms include dyspnea, chest pain, and syncope. Arrhythmias, signs of heart failure, and cardiac tamponade have also been reported [9]. The disease has a high mortality, and treatment typically involves chemotherapy or chemotherapy combined with surgery in patients experiencing hemodynamic compromise [10].

HHV8-positive DLBCL is a rare lymphoma with an incidence of only ~0.1% and is usually associated with HIV infection. The presence of HHV8-positive multicentric Castleman disease (MCD) increases the risk of HHV8-positive DLBCL by approximately 15-fold [11]. It is proposed that increased large plasmablastic-like cells arise in MCD and expansion leads to DLBCL. Of note, in the current case, sampled uninvolved pulmonary lymph node showed a sinusoidal and capsular plasma cell expansion with polytypic light chain expression by kappa/lambda in situ hybridization and scattered HHV8 positive cells, but no definitive demonstration of MCD in the limited lymph node sampling. HHV8-positive DLBCL is an aggressive disease, often involving lymph nodes or spleen, but also may involve the peripheral blood and bone marrow, or disseminate to other visceral organs including the liver, lungs, and gastrointestinal tract. Patients are typically male, and the median age is 47 (with a range of 39-66). [12] Morphologically, the cells often are characterized by a plasmablastic appearance with multiple, eccentrically placed nucleoli. In contrast to PEL, HHV8-positive DLBCL is characteristically negative for EBV and CD138 [13]. Cardiac involvement by HHV8-positive diffuse large B-cell lymphoma is extremely rare, with only one case reported in literature. In that case, a 59-year-old HIV-positive patient presented with fever, night sweats, and a left atrial mass [14]. However, no details on the immunophenotype of the lymphoma other than HHV8-positivity were provided.

Other aggressive lymphomas such as PEL and plasmablastic lymphoma (PBL) can be seen with increased frequency in the context of HIV infection. PEL usually presents as a serous effusion without a distinct tumor mass [15]. Morphologically, the cells have an immunoblastic to plasmablastic appearance with prominent nucleoli and abundant basophilic cytoplasm. They typically express plasma cell-related markers without B-cell antigen expression and are characteristically dual positive for HHV8 and EBV, particularly in the setting of HIV infection. However, the neoplastic cells in the extracavitary variant can show less plasma cell marker expression with increased B-cell antigen expression. In rare cases, PEL may involve the heart, with one case report in the literature describing PEL involving the atria in a 31-year old patient with HIV also with a concurrent large pericardial effusion [16]. In contrast, PBL typically presents with an extranodal mass, most often in the oral cavity or gastrointestinal tract. The neoplastic cells show a plasma cell immunophenotype, with expression of CD138, CD38, MUM1, variable CD79a, and restricted cytoplasmic light chains. EBV is positive, particularly when associated with HIV infection, and HHV8 is consistently absent. Overall, these aggressive lymphomas including HHV8-positive DLBCL, PEL, and PBL can show overlapping features, but the presence or absence of HHV8 and EBV in addition to the relative expression of plasma cell markers or B-cell antigens is helpful in distinguishing between these entities [3, 4] (Table 1).

Herein, we present an unusual case of cardiac involvement by HHV8-associated aggressive lymphoma in a male with long-standing untreated HIV infection diagnosed at autopsy. The pattern of disease, including disseminated lymphadenopathy and lack of effusions, and phenotype with lack of CD138 expression and EBV supports the diagnosis of HHV8-positive DLBCL. Untreated HIV has become much less common with the widespread adoption of ART, however, HIV viremia and reduced CD4-T cell counts predispose to rare and aggressive hematolymphoid malignancies.

## Figures and Tables

**Table 1:** A comparison of the typical immunophenotype and viral association in HHV8-positive DLBCL, PEL, and PBL.

	CD45	CD20	CD19	CD79a	MUM1	CD138	Ig	EBV	HHV8
HHV8-positive DLBCL	+/-	+/-	+/-	—	+	—	+ (c)	—	+
Primary effusion lymphoma	+	—	—	—	+	+	- (c/s)	+	+
Plasmablastic lymphoma	—	—	—	+/-	+	+	+ (c)	+	—

Ig indicates immunoglobulin expression, with c/s indicating cytoplasmic or surface. EBV indicated Epstein-Barr virus in situ hybridization.

## Data Availability

Data sharing not applicable to this article as no datasets were generated or analyzed during the current study.
